# Surface charge changes in spike RBD mutations of SARS-CoV-2 and its variant strains alter the virus evasiveness *via* HSPGs: A review and mechanistic hypothesis

**DOI:** 10.3389/fpubh.2022.952916

**Published:** 2022-08-24

**Authors:** Zhongyun Zhang, Juan Zhang, Jiqiu Wang

**Affiliations:** ^1^Department of Endocrine and Metabolic Diseases, Shanghai Institute of Endocrine and Metabolic Diseases, Ruijin Hospital, Shanghai Jiao Tong University School of Medicine, Shanghai, China; ^2^Shanghai National Clinical Research Center for Metabolic Diseases, Key Laboratory for Endocrine and Metabolic Diseases of the National Health Commission of the PR China, Shanghai National Center for Translational Medicine, Ruijin Hospital, Shanghai Jiao Tong University School of Medicine, Shanghai, China

**Keywords:** SARS-CoV-2, hACE2, HSPGs, RBD, point mutation, variant strain, electrostatic interaction

## Abstract

With the COVID-19 pandemic continuing, more contagious SARS-CoV-2 variants, including Omicron, have been emerging. The mutations, especially those that occurred on the spike (S) protein receptor-binding domain (RBD), are of significant concern due to their potential capacity to increase viral infectivity, virulence, and breakthrough antibodies' protection. However, the molecular mechanism involved in the pathophysiological change of SARS-CoV-2 mutations remains poorly understood. Here, we summarized 21 RBD mutations and their human angiotensin-converting enzyme 2 (hACE2) and/or neutralizing antibodies' binding characteristics. We found that most RBD mutations, which could increase surface positive charge or polarity, enhanced their hACE2 binding affinity and immune evasion. Based on the dependence of electrostatic interaction of the epitope residue of virus and docking protein (like virus receptors or antibodies) for its invasion, we postulated that the charge and/or polarity changes of novel mutations on the RBD domain of S protein could affect its affinity for the hACE2 and antibodies. Thus, we modeled mutant S trimers and RBD-hACE2 complexes and calculated their electrotactic distribution to study surface charge changes. Meanwhile, we emphasized that heparan sulfate proteoglycans (HSPGs) might play an important role in the hACE2-mediated entry of SARS-CoV-2 into cells. Those hypotheses provide some hints on how SARS-CoV-2 mutations enhance viral fitness and immune evasion, which may indicate potential ways for drug design, next-generation vaccine development, and antibody therapies.

## Introduction

Severe acute respiratory syndrome coronavirus 2 (SARS-CoV-2) led to an unprecedented pandemic known as Coronavirus disease 2019 (COVID-19), which is raging world widely, resulting in catastrophic effects on human health and a terrible social as well as a financial crisis (1, 2). According to World Health Organization (WHO; https://www.who.int), 494.6 million infections of SARS-CoV-2 and at least 6.2 million deaths were confirmed till to 8 April 2022. Unfortunately, the pandemic is continuing. It is much likely that SARS-CoV-2 will coexist with humans over a long period (3). Unfortunately, no effective treatment for COVID-19 has been emerging in the past 2 years, especially for critical patients (3). Very recently, an oral protease inhibitor named Paxlovid drug has been emergently approved by food and drug administration (FDA) to treat the virus (4, 5). In the context of the recurrent pandemic, effective vaccination is still warranted for preventing the further spread of infection and slowing down the progression of the disease (6).

**Figure 1:**
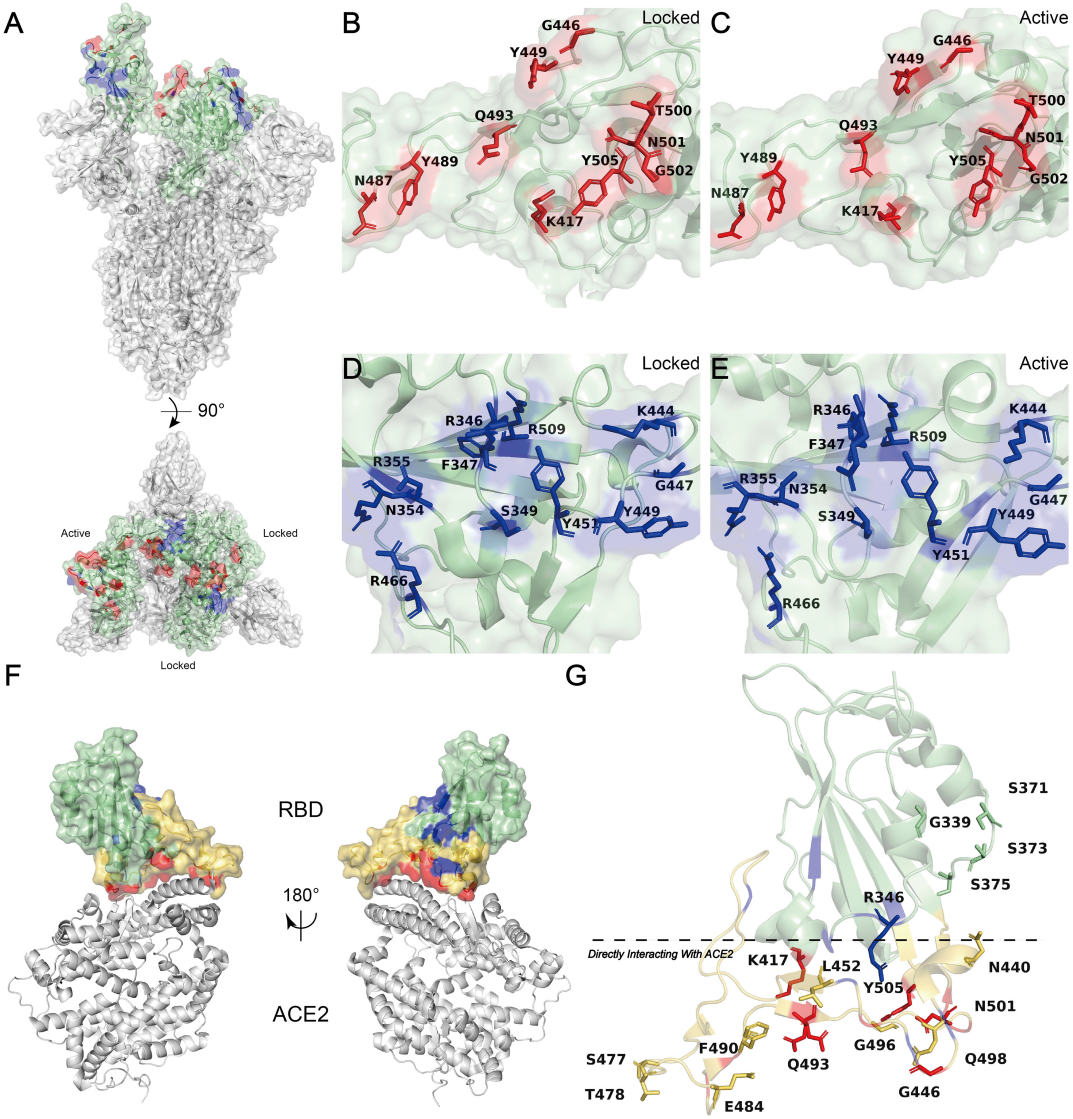
Molecular Modeling of the SARS-CoV-2 S RBD. **(A)** Side (up) and top (down) views of the molecular model of SARS-CoV-2 S trimer (PDB: 7DWZ) rendered with PyMOL (Color code: green, RBD; red, hACE2 binding sites; blue, HSPGs binding sites) (10). **(B,C)** The hACE2 binding residues (K417, G446, Y449, N487, Y489, Q493, T500, N501, G502, and Y505) of RBD are colored red and shown with their side chains (11). **(B)** Locked configuration. **(C)** Active configuration. **(D,E)** The HSPG binding residues (R346, F347, S349, N354, R355, K444, G447, Y449, Y451, R466, and R509) of RBD are colored blue and shown with their side chains (19). **(D)** Locked configuration. **(E)** Active configuration. **(F)** Front (right) and back (left) views of the molecular model of RBD-hACE2 complex (PDB: 6M0J) rendered with PyMOL (Color code: green, RBD; orange-yellow, RBM; red, hACE2 binding sites; blue, HSPGs binding sites) (11). **(G)** Positions of 18 RBD Mutation sites described in Table 1 (PDB: 6M0J) labeled with PyMOL) (11). Residues below the black line are at the direct hACE2 binding interface (Mainly including RBM and K417). Mutants located in the hACE2 binding sites are highlighted in red and RBM is in orange-yellow.

According to the COVID-19 vaccine candidates' solution devised by WHO, the research and development scheme of the vaccine primarily focused on spike (S) protein, a glycosylated trimer that protrudes from the SARS-CoV-2 lipid envelope (7, 8), since two important processes of SARS-CoV-2 entering host cells, including receptor identification and subsequent membrane fusion, are mainly mediated by S protein. The ectodomain (including S1 and S2 domains) along with the transmembrane domain and the intracellular cytoplasmic domain together form the S protein (7, 9, 10). Broken down further, the distal S1 domain comprises an N-terminal domain (NTD, residues 13 to 304) and a receptor-binding domain (RBD, residues 319 to 541), while the membrane-anchored S2 domain, also known as the C-terminal domain (CTD), contains the fusion machinery (Figure 1A) (9, 10). RBD can combine with the entry-receptor called human protease angiotensin-converting enzyme 2 (hACE2), which could prime transmembrane protease serine type2 (TMPSS2) to cleavage and activate S protein, leading to the membrane fusion and subsequent genetic material released from SARS-CoV-2 into the host cells' cytoplasm (Figures 1B,C) (11, 12). What's more, RBD-hACE2 interacted interface is the hydrophilic network, which contains 13 hydrogen (H) bonds as well as 2 salt bridges (11). A SARS-CoV-2 crystal structure research indicated that a receptor-binding motif (RBM, residues 437 to 508), which mediates contacts with hACE2, is contained in the RBD (Figure 1F) (11, 13). Besides, a unique residue (Lys417) outside the RBM forms salt-bridge interaction with hACE2 (Figure 1G) (11). Interestingly, George et al. identified the presence of a prion-like domain (PrD, residues 473 to 510), with the ability to switch rapidly among multiple conformations, in the RBD of SARS-CoV-2 plays an important role in the tight connection between RBD-hACE2 (14). Blocking the binding and fusion between S protein and host cells by neutralization antibodies theoretically prevents virus infection, and on the other hand, strengthening the binding or fusion by certain mutations of the virus could promote its infection (15).

**Figure 2:**
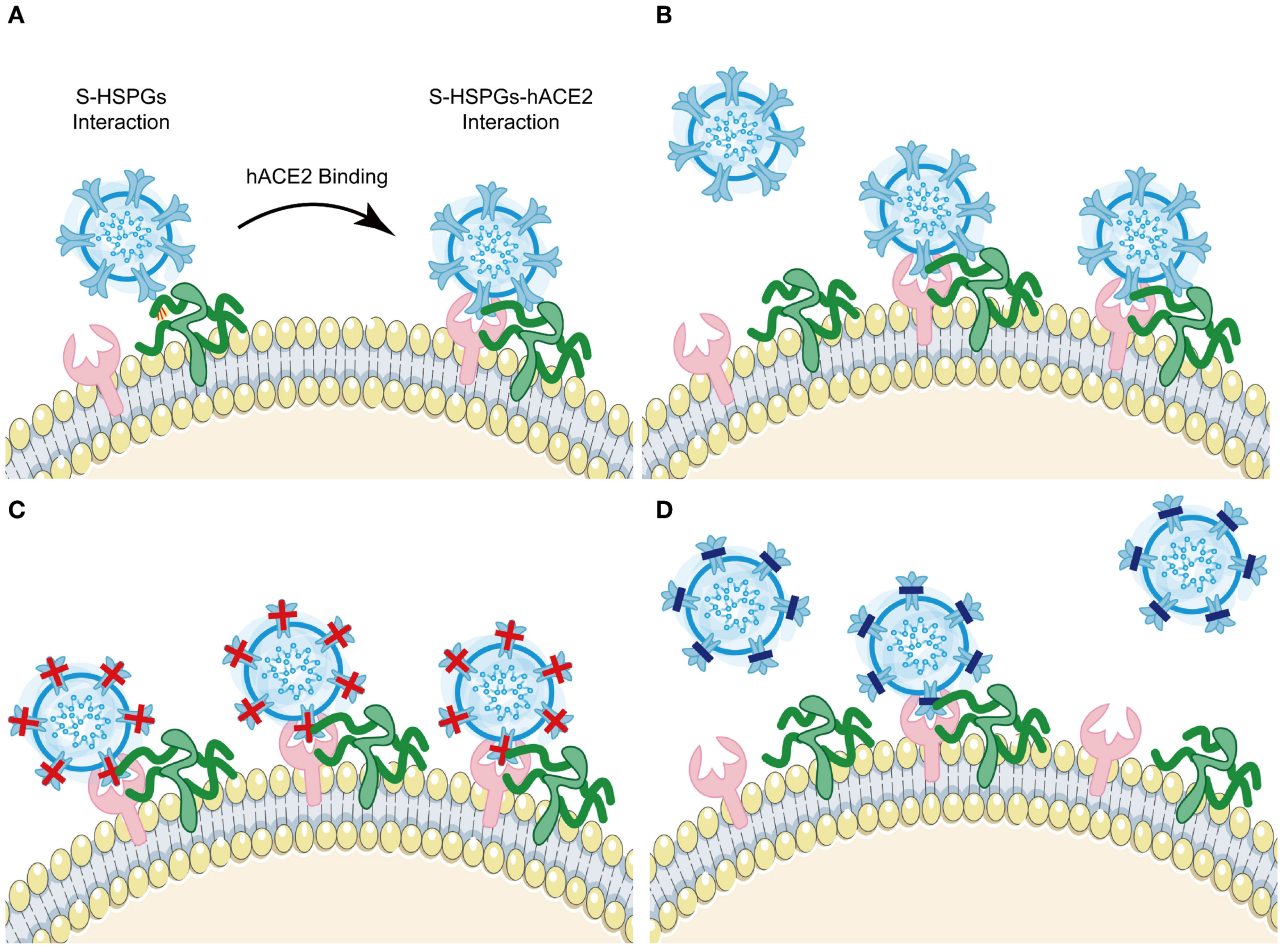
HSPGs enhances SARS-CoV-2 S protein binding to hACE2 and potentiates viral infection. **(A)** Schematic diagram showing HSPGs-led activate RBD conformation and subsequent S-HSPGs-hACE2 interaction (Color code: blue, SARS-CoV-2; pink, hACE2; green, HSPGs). **(B)** Schematic model depicts normal SARS-CoV-2 recognition and entry. **(C)** Schematic model depicts SARS-CoV-2 variants (like E484K), whose surface charge is increasingly positive, recognition and entry. **(D)** Schematic model depicts SARS-CoV-2 variants (like K417N/T), whose surface charge is increasingly negative, recognition and entry.

Looking deep into nature, the attachment and following interaction of the S protein to cellular heparan sulfate (HS) is the initial phase of the viral invading the target cell (16–18). HS is a negative charge-enriched linear and sulfated polysaccharide molecule (19). As part of the heparan sulfate proteoglycans (HSPGs), HS adhered to a small fraction of proteoglycans, which are ubiquitous on cell surfaces and in the extracellular matrix (ECM) (19–21). There is a strong interaction between negatively charged HSPGs and positively charged amino acid residues (Arg346, Arg355, Lys444, Arg466, and probably Arg509) located in RBD (19). This electrostatic interaction facilitates the conformational transition of the RBD from an inactive (closed) state to an active (open) state, thus supporting and enhancing RBD-hACE2 binding simultaneously (Figures 1D,E, 2A) (19). Moreover, research suggested that the other six RBD amino acids (Phe347, Ser349, Asn354, Gly447, Tyr449, and Tyr451) form H-bonding interactions with HSPGs to stabilize the association (19).

It is hugely encouraging to see 36 COVID-19 vaccines developed from different strategies have been accepted and deployed globally (https://covid19.trackvaccines.org/vaccines/), which is critical to blocking the pandemic of COVID-19 (22). However, due to constant evolution and selection pressure, many new novel SARS-CoV-2 variants, particularly variants of concern (VOCs), recently emerged with different antigenic properties compared to the wild-type (WT) (23). It's remarkable that the B.1.1.7+Q.^*^ (Alpha) lineage was first identified in Britain and quickly dominated (24). Meanwhile, the B.1.351 (Beta) lineage in South Africa (25), the P.1 (Gamma) lineage in Brazil (26), the B.1.617.2+AY.^*^ (Delta) lineage in Indian (27), and the B.1.1.529+BA.^*^ (Omicron) lineage in South Africa (28) have raised serious risk, perhaps due to many reasons, such as enhanced infectivity, high pathogenicity, and immune evasion. Especially Omicron, owing 15 RBD substitutions, has now out competed pre-existing lineages and become the dominant worldwide (28). Since the S protein is critical for SARS-CoV-2 entering into the target cells by interacting with the viral receptor hACE2 and HSPGs, mutations inside RBD are highly concerned for their potential worsening in viral invasiveness and immune evasion.

## Hypothesis

Changes in the electrostatic interactions between mutations and HSPGs indeed contribute to the occurrence and development of some diseases, like in type III hyperlipoproteinemia and Alzheimer's disease (AD) (29, 30). For instance, variants (E327G/A) of the dengue virus type 4 (DENV-4) that acquired a relatively positive surface charge facilitated interactions to gain an entry into target cells, by reducing their inability to bind to negatively charged glycosaminoglycans (31). Based on these pieces of evidence and correlational studies (19, 29–32), we hypothesize that mutations inside RBD may enhance the physical binding of S protein to HSPGs by increasing surface positive charge and polarity, increasing the local concentrations and the dwelling course of the virus, yielding more chances of the molecular collision between S protein and hACE2, and eventually lead to more virus invasiveness (Figure 2B). As RBD is the primary recognized target of virus-neutralizing antibodies, these RBD mutants may escape control by both vaccine-induced and convalescent immune responses by the similar mechanisms if the antibodies do not target to block the mutant residue (33, 34).

## Results

**Figure 3:**
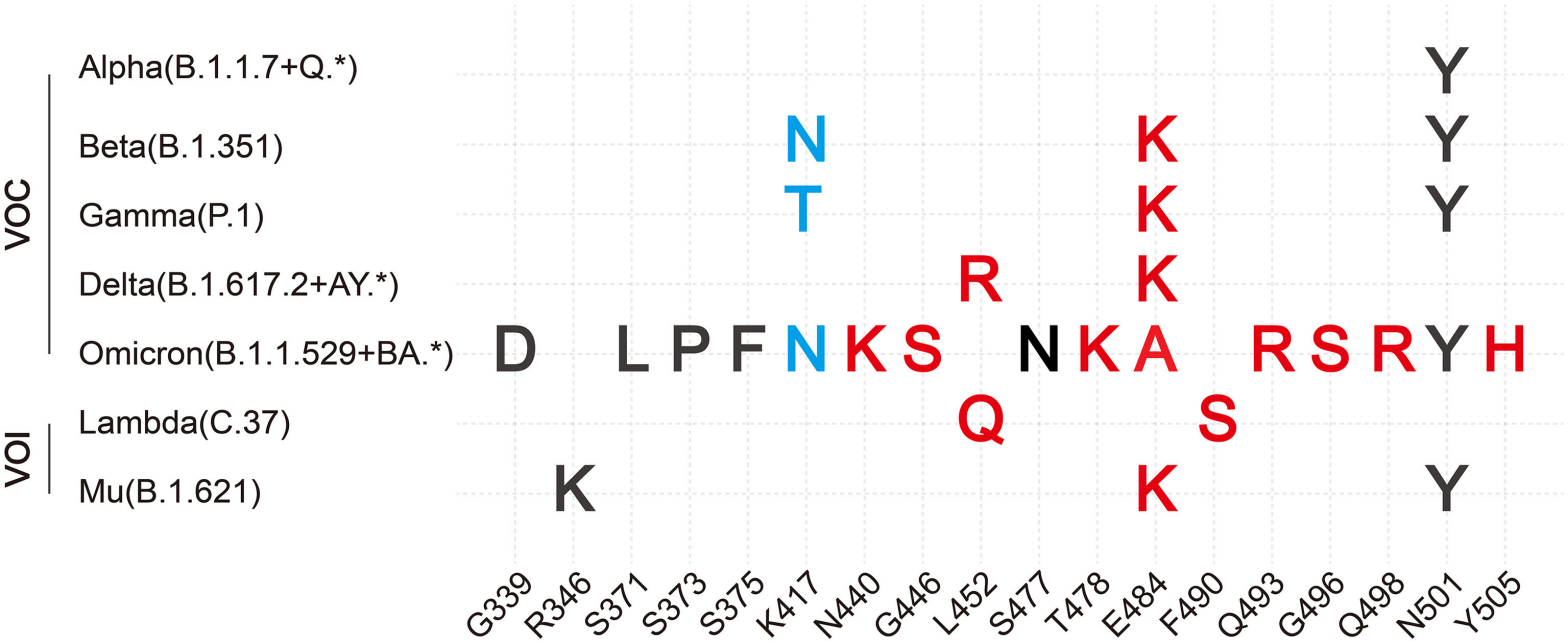
Summary of VOCs and VOIs' most common mutations in RBD. Description of VOCs and VOIs' most common mutations in the RBD domain of S protein combined with our hypothesis. Here, only changes of binding affinity between S and hACE2 caused by RBD mutations are considered. Both red and blue colors are support our hypothesis. Red color represents the positive-going charge and (or) polarity changes that will strengthen the binding affinity, while the blue color shows the negative-going charge and (or) polarity changes that will weaken the binding affinity. The black color indicates that our hypothesis has not been denied yet based on present data of these mutations, perhaps because their residue positions do not directly interact with hACE2 or amino acid changes are of the same type.

Several RBD mutations after natural selection have been shown to affect viral infectivity, pathogenic mechanism, and immune evasion. Here, we concluded 21 mutations in the RBD domain of VOCs and Variants of Interest (VOIs) and summarized their change of hACE2 binding affinity and ability to escape protective antibodies respectively (Table 1, Figure 3). Among these mutations, eleven mutations (G339D, K417N, K417T, N440K, L452R, T478K, E484A, E484K, Q493R, Q498R, and Y505H) have charge changes, seven mutations (S371L, S373P, S375F, G446S, L452Q, F490S, and G496S) have polarity changes, and the amino acid changes of three mutations (R346K, S477N, and N501Y) are of the same type.

**Table 1:** Summary of VOCs and VOIs' most common mutations in the RBD domain of S protein.

**Mutations**	**Pango lineage**	**Location of first identification**	**Change of amino acids**		**hACE2 binding affinity**	**Ability to escape protective antibodies**
			**Before**	**After**		
**G339D** (41, 56)	B.1.1.529+BA.^*^	South Africa	Non-polar	Negative	No significant change	Increase
**^#^R346K** (42, 43)	B.1.621	Colombia	Positive	Positive	No significant change	Increase
**S371L** (41, 56)	B.1.1.529+BA.^*^	South Africa	Uncharged Polar	Non-polar	No significant change	Increase
**S373P** (41, 56)	B.1.1.529+BA.^*^	South Africa	Uncharged Polar	Nonpolar	No significant change	Increase
**S375F** (41, 56)	B.1.1.529+BA.^*^	South Africa	Uncharged Polar	Non-polar	No significant change	Increase
^*^K417N (15, 25)	B.1.351& B.1.1.529+BA.^*^	South Africa	Positive	Uncharged Polar	Decrease	Increase
^*^K417T (15)	P.1	Brazil	Positive	Uncharged Polar	Decrease	Increase
**N440K** (56, 57)	B.1.1.529+BA.^*^	South Africa	Uncharged Polar	Positive	Increase	Increase
^*^G446S (44, 56)	B.1.1.529+BA.^*^	South Africa	Non-polar	Uncharged Polar	Increase	Increase
**L452R** (35, 51)	B.1.617.2+AY.^*^	India	Non-polar	Positive	Increase	Increase
**L452Q** (45, 58)	C.37	Peru	Non-polar	Uncharged Polar	Increase	Increase
**S477N** (15, 56)	B.1.1.529+BA.^*^	South Africa	Uncharged Polar	Uncharged Polar	Increase	Increase
**T478K** (36, 52)	B.1.617.2+AY.^*^ & B.1.1.529+BA.^*^	India & South Africa	Uncharged Polar	Positive	Increase	Increase
**E484A** (56, 59)	B.1.1.529+BA.^*^	South Africa	Negative	Non-polar	Increase	Increase
**E484K** (25, 53, 54)	B.1.351&P.1& B.1.617.2+AY.^*^ & B.1.621	South Africa & Brazil & Colombia	Negative	Positive	Increase	Increase
**F490S** (46, 60)	C.37	Peru	Non-polar	Uncharged Polar	Increase	Increase
^*^Q493R (56, 61)	B.1.1.529+BA.^*^	South Africa	Uncharged Polar	Positive	Increase	Increase
**G496S** (47, 56)	B.1.1.529+BA.^*^	South Africa	Non-polar	Uncharged Polar	Increase	Increase
**Q498R** (47, 56)	B.1.1.529+BA.^*^	South Africa	Uncharged Polar	Positive	Increase	Increase
^*^N501Y (55, 62)	B.1.1.7 + Q.^*^ & B.1.351 & P.1 & B.1.1.529 + BA.^*^ & B.1.621	Britain & South Africa & Brazil & Colombia	Uncharged Polar	Uncharged Polar	Increase	Increase
^*^Y505H (56, 59)	B.1.1.529+BA.^*^	South Africa	Uncharged Polar	Positive	Increase	Increase

# HSPGs-binding residues;

* hACE2-binding residues.

### RBD mutations affect the binding affinity to hACE2 or HSPGs

As noted earlier, RBM interacts directly with hACE2 and contains most of the hACE2 contact residues. What's more, Lan et al. found that ten RBD residues (Lys417, Gly446, Tyr449, Asn487, Tyr489, Gln493, Thr500, Asn501, Gly502, and Tyr505) are responsible for hACE2 identification and interaction by 13 H bonds and 2 salt bridges (11). It is worth mentioning that Lys417 is a unique residue outside the RBM, which forms salt-bridge interaction with hACE2 (11). So, this article focused on the mutations in Lys417 and RBM residues. According to the experimental assessment, most of these mutations, including L452R, T478K, and E484K/Q, strengthened the binding affinities between RBD and hACE2, while the RBD-hACE2 binding affinities of exceptional mutations K417N/T were weakened (15, 25, 35, 36). The common feature of former mutations belongs to the substitutions of uncharged (or negative-charged) residues to positive ones, and the latter belongs to positive to uncharged. Negatively charged sulfate groups in HSPGs can bind and neutralize positively-charged residues of target proteins, which provides a multivalent landing plug for proteins through electrostatic interaction (37, 38). For SARS-CoV-2, HSPGs acts as a coreceptor activating the S protein for interacting with hACE2, eventually leading to increased odds of virus invasiveness. The variant B.1.617 bearing the D614G (negatively charged amino acid Asp to uncharged nonpolar amino acid Gly) substitution has been identified in Maharashtra, India, which is mainly characterized by an alteration in the electrostatic potential on the surface of RBD (39). In subline B.1.617.1 (Kappa), the uncharged nonpolar amino acid Leu located at the 452 site turns to the positively charged amino acid Arg (L452R), and the negatively charged amino acid Glu located at the 484 position is substituted by the uncharged polar amino acid Gln (E484Q). In addition to the same L452R mutation, the B.1.617.2 (Delta) sublineage bears another mutation at position 478 where the uncharged polar amino acid Thr is replaced by the positively charged amino acid Lys (T478K). In brief, the substitutions in B.1.617 variants increase the positive electrostatic potential on the S trimers surface. At this juncture, we assume that the L452R, T478K, and E484Q substitutions enhance virus-HSPGs interactions, which promote the combining capacity between S protein and hACE2 receptor (Figure 2C). The same assumption applies to E484K. Like two sides of a coin, the amino acid residues substitution at 417 residue from the positively charged Lys (K) to a polar but uncharged Asn/Thr (N/T) is predicted to decrease the positive electrostatic potential surface, which reduced the interaction probability between S RBD and HSPGs. In addition, Cheng et al. have also reported that the salt bridge formed between hACE2 and RBD was disrupted by the K417N/T mutation (40). Hence, the hACE2 binding affinity of the K417N/T variant was decreased (Figure 2D) (15). Both sides seem to support the hypothesis. However, the locations of G339D, R346K, S371L, S373P, and S375F are relatively far from the binding interface of hACE2 and RBD. Although Arg346 is one of the HSPGs-binding sites, both Arg(R) and Lys(K) are positively charged residues. So, no significant change in the hACE2 binding affinity of these 5 mutations can be explained (41–43).

Furthermore, G446S, L452Q, F490S, and G496S, whose common features are mutating the hydrophobic residues (Gly, Leu, and Phe) to polar residues (Ser and Gln), enhance the interactions with hACE2 (44–47). This high binding affinity can be attributed to the increased hydrogen bonding interface of RBD-HSPGs and/or increased hydrophilic contact at the interfaces of RBD-hACE2.

### RBD mutations affect surface electrostatic potential of SARS-CoV-2

**Figure 4:**
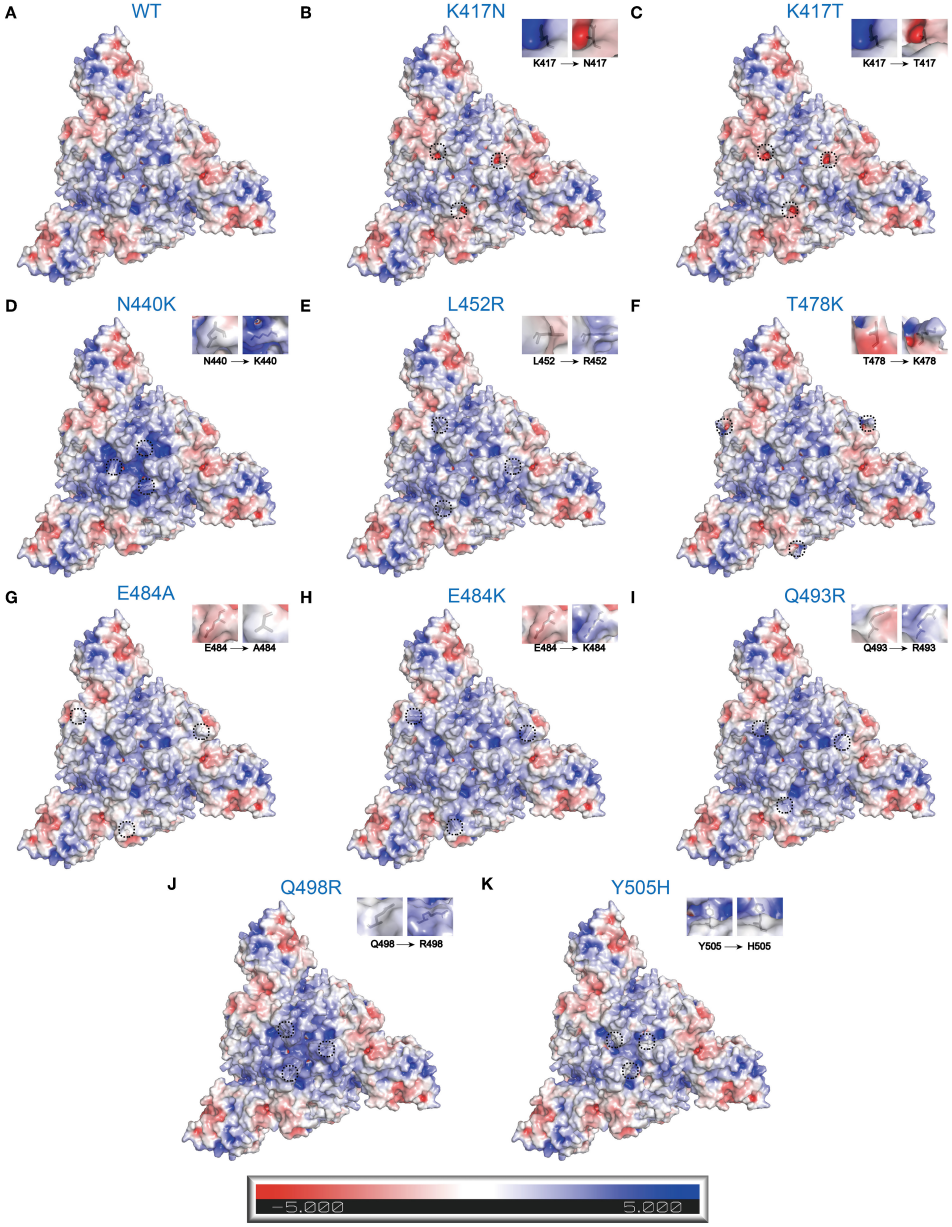
Electrostatic charge distribution of WT and RBD single point mutated SARS-CoV-2 S trimers (PDB: 7DF3) (70). The molecular surface was calculated by PyMOL plugin APBS electrostatics with default settings and colored based on the molecular electrostatic potential in a gradient from red (negative, −5.0kT/e) to blue (positive, +5.0kT/e). The mutated side chains are marked in black color. The black dashed circle indicates the location of mutations. **(A)** WT, **(B)** K417N, **(C)** K417T, **(D)** N440K, **(E)** L452R, **(F)** T478K, **(G)** E484A, **(H)** E484K, **(I)** Q493R, **(J)** Q498R, and **(K)** Y505H.

Protein electrostatic properties depend on the whole and partial charge distribution of the three-dimensional protein structure (48). Electrostatic interactions are crucial to many protein-protein/ligand interactions. To better describe electrostatic changes in mutant proteins, we used the Poisson-Boltzmann equation to calculate electrostatic potential on the surfaces of WT and mutant S trimers (Figure 4). Here, we only concentrated on the point mutations involving changes in charge. Single-point mutations have significant effects on electrostatic distribution, especially at substitutive sites and their immediate vicinity. Compared to WT S trimer (Figure 4A), K417N/T, whose amino acids change from the positive (Lys) to the uncharged polar (Asn and Thr), are predicted to decrease the S-trimers' electrostatic potential to a more negative surface (Figures 4B,C). While N440K, L452R, T478K, Q493R, Q498R, and Y505H, whose amino acids change from uncharged polar (Asn, Thr, Gln, and Tyr) or Nonpolar (Leu) to positive (Lys, Arg, and His), are estimated to increase the S-trimers' electrostatic potential to a more positively charged surface (Figures 4D–F,I–K). This situation also applies to E484A/K, whose amino acids change from negative (Glu) to Nonpolar (Ala) or positive (Lys) (Figures 4G,H).

**Figure 5:**
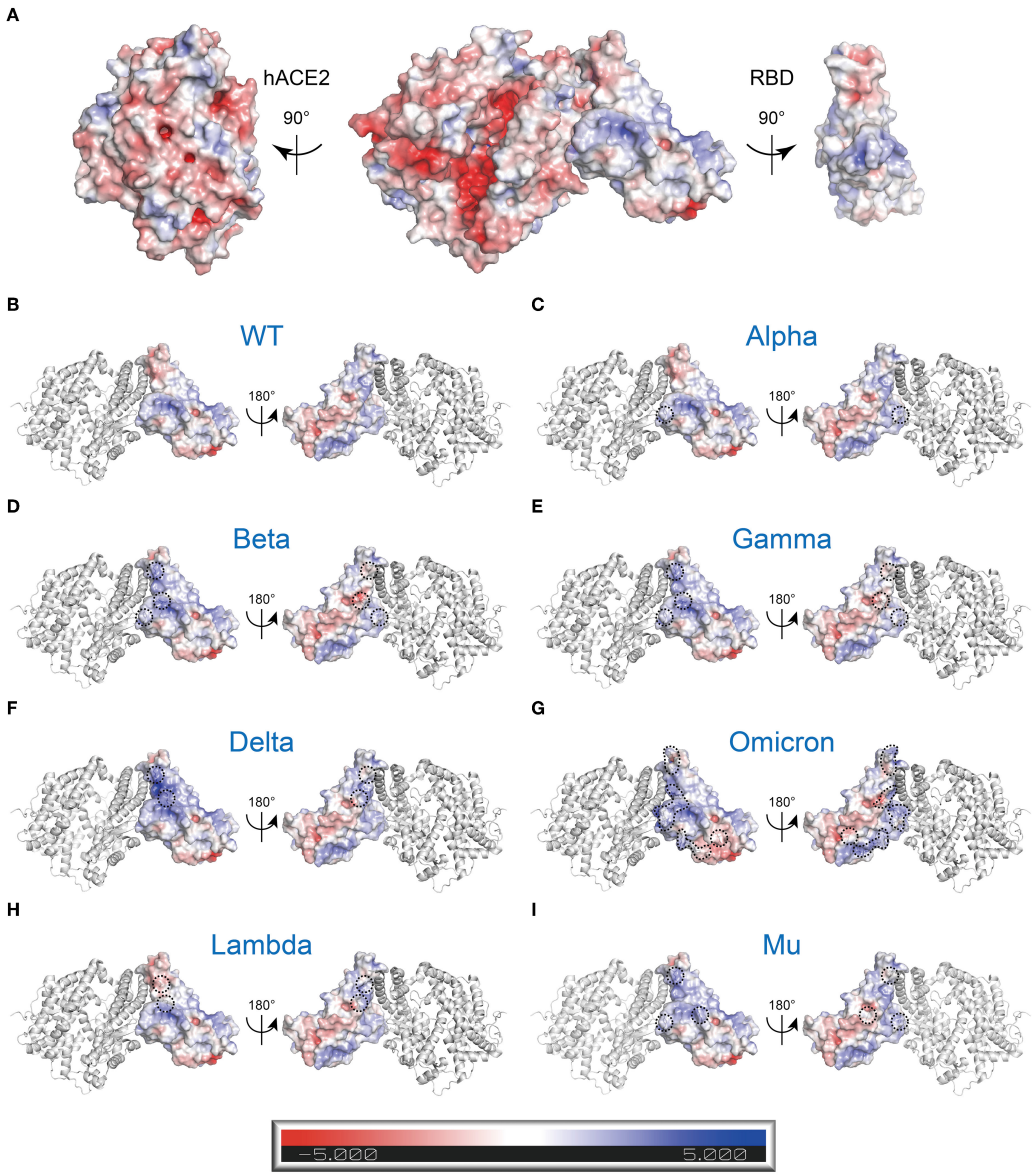
Electrostatic charge distribution of WT and mutant RBD-hACE2 complexes (PDB: 6M0J) (11). The molecular surface was calculated by PyMOL plugin APBS electrostatics with default settings and colored based on the molecular electrostatic potential in a gradient from red (negative, −5.0kT/e) to blue (positive, +5.0kT/e). The mutated side chains are marked in black color. The black dashed circle indicates the location of mutations. **(A)** Electrostatic properties of the WT RBD-hACE2 complex. Left: RBD binding interface in hACE2, Middle: RBD-hACE2 complex, Right: hACE2 Binding Interface in RBD. **(B-I)** Electrostatic properties of the WT and mutant RBDs of RBD-hACE2 complexes. hACE2 (gray) is included as a reference. **(B)** WT, **(C)** Alpha (B.1.1.7+Q.*; N501Y), **(D)** Beta (B.1.351; K417N, E484K, and N501Y), **(E)** Gamma (P.1; K417T, E484K, and N501Y), **(F)** Delta (B.1.617.2+AY.*; L452R, and E484K), **(G)** Omicron (B.1.1.529+BA.*; G339D, S371L, S373P, S375F, K417N, N440K, G446S, S477N, T478K, E484A, Q493R, G496S, Q498R, N501Y, and Y505H), **(H)** Lambda (C.37; L452Q and F490S), and **(I)** Mu (B.1.621; R346K, E484K, and N501Y).

Additionally, adaptive Poisson-Boltzmann equation solver (APBS) analysis of the RBD-hACE2 complex showed charge distribution appears to vary between RBD and hACE2. The RBD surface that faces the hACE2 appears positively charged surface, while the hACE2 surface that faces the RBD has a complementary negative charged surface (Figure 5A). Besides, the region around R346-F347-S349-N354-R355-K444-G447-Y449-Y451-R466-R509 residues in RBD displays powerful positive electrostatic potentials, which is matched by the negatively charged HSPGs (Figure 5A) (19). It is easy to speculate that charge–charge complementary interaction could be crucial for RBD engaging with hACE2 or HSPGs.

**Figure 6:**
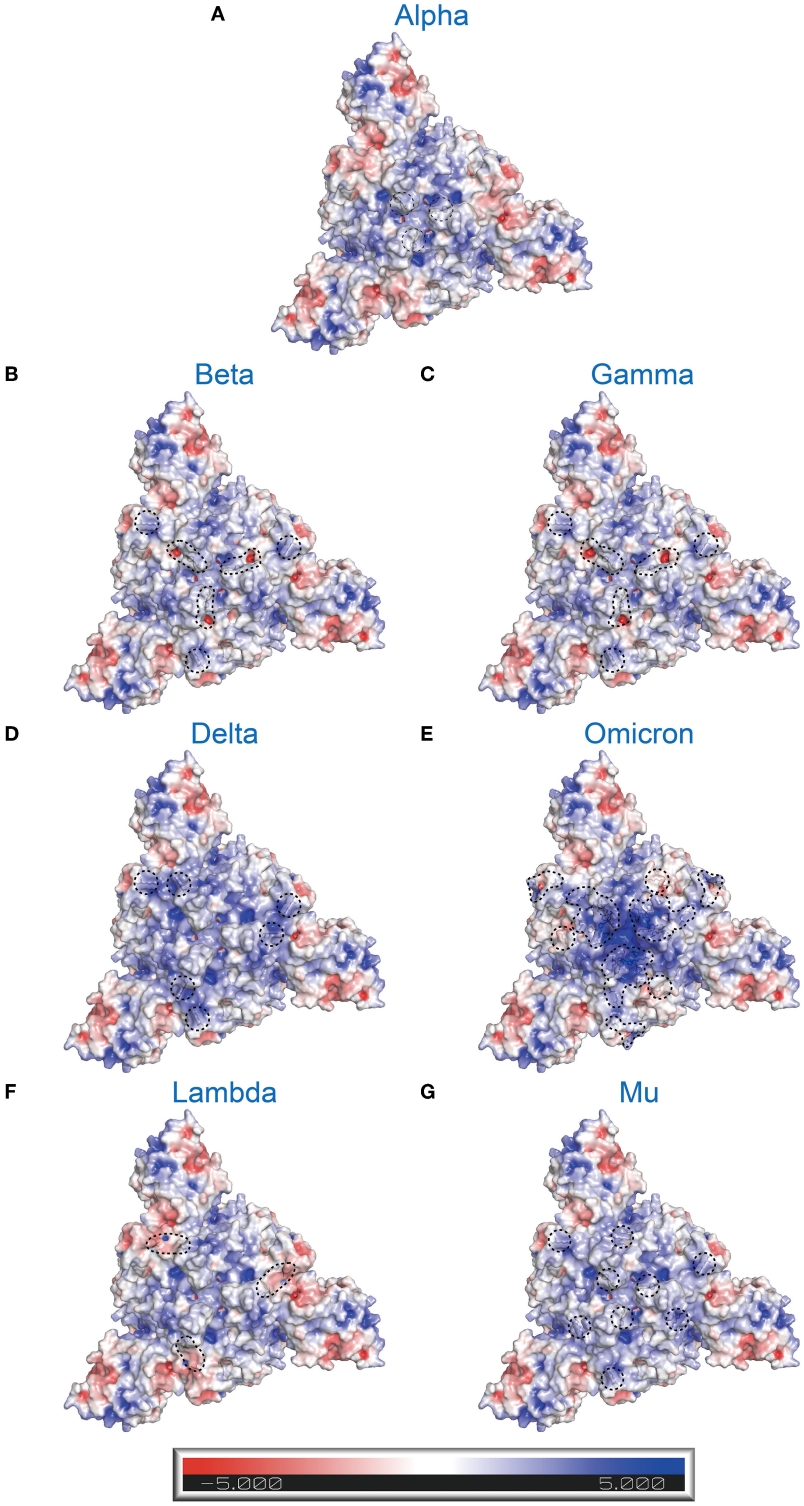
Electrostatic charge distribution of SARS-CoV-2 variants' S trimers (only consider RBD mutations; PDB: 7DF3) (70). The molecular surface was calculated by PyMOL plugin APBS electrostatics with default settings and colored based on the molecular electrostatic potential in a gradient from red (negative, −5.0kT/e) to blue (positive, +5.0kT/e). The mutated side chains are marked in black color. The black dashed circle indicates the location of mutations. **(A)** Alpha (B.1.1.7+Q.*; N501Y), **(B)** Beta (B.1.351; K417N, E484K, and N501Y), **(C)** Gamma (P.1; K417T, E484K, and N501Y), **(D)** Delta (B.1.617.2+AY.*; L452R and E484K), **(E)** Omicron (B.1.1.529+BA.*; G339D, S371L, S373P, S375F, K417N, N440K, G446S, S477N, T478K, E484A, Q493R, G496S, Q498R, N501Y, and Y505H), **(F)** Lambda (C.37; L452Q and F490S), and **(G)** Mu (B.1.621; R346K, E484K, and N501Y).

It is widely known that different variants have some common and unique mutations. How does this affect the whole electrostatic distribution of protein, when a single RBD point mutation is combined with other co-occurring RBD mutations? Hence, the surface electrostatic potential distributions of the S trimers and RBDs of VOCs and VOIs were also computed (Figures 5B–I, 6). The most striking was Omicron, eleven (K417 N, N440K, G446S, S477 N, T478K, E484A, Q493K, G496S, Q498R, N501Y, and Y505H) of its fifteen RBD mutations are located at RBD-hACE2 interface. Compared to WT RBD, the same interface in Omicron RBD has larger patches with positive potential (Figures 5E, 6G). These results may help us understand how specific mutations lead to RBD dysfunction by altering electrostatic potential.

### RBD mutations affect antibody binding ability of SARS-CoV-2

Mutations can facilitate immune escape *via* altering the antigenic properties of S trimers by various distinct mechanisms (49). The physical and biological features of epitopes inevitably changed due to amino acid substitutions, which potentially attenuate or even abolish antibody neutralization of vaccines. For instance, the E484K (from negatively charged Glu to positively charged Lys) shows the potential to weaken antibody binding (49). This may also apply to other point mutations, including G339D, K417N/T, N440K, L452R, T478K, E484A/K, Q493R, Q498R, and Y505H, which are similarly accompanied by charge changes in amino acid substitutions. Meanwhile, glycoprotein substitutions with strong membrane-receptor binding affinity may facilitate immune escape *via* shifting the binding balance of glycoprotein and neutralizing antibodies (50). The S amino acid substitutions, including N440K, G446S, L452R/Q, S477N. T478K, and E484A/K, F490S, Q493R, G496S, Q498R, N501Y, and Y505H, increase hACE2-binding affinities, which potentially serve as a means of immune evasion (15, 25, 35, 36, 44–47, 51–62). In addition, these substitutions may also disrupt antibody neutralization in a manner of altering the protein conformation, so that virus antigenic epitopes are differently displayed. In short, the hypothesis hints that the immune escape of mutations is also associated with the obvious alteration of surface charge and polarity.

## Discussion

The COVID-19 pandemic has been ongoing for more than 2 years. After a short period of a flattening curve, a new wave of infection presents in the United Kingdom, which usually precedes the world spread (63). Unfortunately, this occurs in people aged 12 years and older, 79.5% of which have been vaccinated with two doses of anti-SARS-CoV-2 (63). Under the selective pressure of transmissibility, immune evasion, and vaccination, the emergence of novel SARS-CoV-2 VOCs is a high probability event. The virus with increasing transmissivity, disease severity, and resistance to neutralizing antibodies from previous infection or vaccines, together vanning vaccine antibodies in bodies, will make containment of the pandemic more difficult. Mutations, occurring on S and especially on its RBD, are particularly noteworthy due to their crucial role in transmissibility and vaccine effectiveness. Now-a-days, enormous studies on SARS-CoV-2 variants have been carried out rapidly and comprehensively, most of which concentrated on the changes in their hACE2 binding affinity and antigenic change. However, the understanding of basic biophysical features in SARS-CoV-2 variants is still poor, particularly the change of charge of SARS-CoV-2 variants and its coaction with ECM, especially HSPGs, which will be of clinical relevance for curing its invasiveness and immune escape.

According to the circumstantial evidence presented in the published paper, we propose here that the mutations inside RBD of S change virus invasiveness and immune escape, at least in part, relying on biochemical and biophysical alterations, especially for the changes of surface charge. That is to say, if the positive electrostatic potential surface and polarity of the S increase, it will enhance the affinity of S to HSPGs and then the receptor hACE2, thereby potentiating viral infection. Meanwhile, the antigenic changes may lead to immune escape. Apart from that, the tissue tropism of variants might be affected due to charge changes. As noted earlier, hACE2 and TMPSS2 are crucial for SARS-CoV-2 invading cells, and their distribution and expression may determine the main infected part of the respiration system (12). A Real-time Quantitative polymerase chain reaction (qPCR) analysis of human lung tissues suggested that the mRNA expression of hACE2 had a higher trend in the upper-airway tissues (64). In contrast, TMPRSS2's mRNA was higher expressed in the lower-airway tissues (64). Omicron is characterized by a higher hACE2 binding affinity due to a more positively charged surface. Meanwhile, an experiment showed that the entry of Omicron was impaired in high TMPRSS2 expressed cells (64). In conclusion, Omicron might have the propensity to invasive the upper airway rather than the lower airway, which could explain that the disease severity of Omicron is more attenuated compared to previous emerging variants (65). As new mutations continue to emerge, such a hypothesis, if it is valid with experiments or novel variant strains, will lead to precise predictions of variants phenotype and outcome, and it provides insights for immune and other therapies for SARS-CoV-2 infections. Of course, further investigations are still needed to probe and verify the scientific mechanisms assumed in this hypothesis.

To explore this possibility *in vivo*, WT SARS-CoV-2 and mutant SARS-CoV-2 bearing RBD mutations can be applied to infect suitable animal models, which were then treated with or without heparin, and the mean virus intensity in the respiratory mucosal epithelium or other tissues could be quantified. While, *in vitro*, to investigate whether the mutation enhances binding capacity between RBD and hACE2 in a way of HSPGs dependent, we can analyze the binding ability of RBD mutated proteins after treatment of heparinase (HSase), an enzyme that degrades cell surface HSPGs. In addition, at molecular levels, recombinant RBD mutated proteins can be incubated with hACE2 with or without heparin, then comparing the binding complex with western blotting or cryo-electron microscopy (55).

In fact, we note that COVID-19 patients commonly in serious conditions are often accompanied by thrombotic complications. Thus, these patients are conventionally treated with heparin, unfraction heparin (UFH), or low molecular weight heparin (LMWH) mainly as an anticoagulant (66). With the assumption described above, we infer that targeting SARS-CoV-2 binding to HSPGs may potentially interfere with virus infection as structurally defined heparin/UFH/LMWH, together with their mimetics might bind the S protein and function as a competitive inhibitor by competing with cell surface HSPGs to prevent viral adhesion, thus decreasing infectivity. A study found that serum bioavailability of heparin was low by intranasal or inhalation routes (67). Moreover, nasal and tracheobronchial epithelial cells acted as a gateway for initial SARS-CoV-2 infection and spread (68). Considering the results of the two above studies, Tandon et al. proposed a self-administered nasal spray of UFH, which might avoid dangerous complications or severe side effects of anticoagulation therapies (69). More importantly, the use of heparin may have benefits in preventing or treating COVID-19. Given the crucial role of the charge changes in the interaction between mutated S and HSPGs in the onset of COVID-19, heparin or its derivative might be a potential and efficient treatment to mitigate new variants' infection, based on our assumption. However, the administration way, timing, dosage, individual status, and other conditions should be systemically considered in COVID-19 patients treated with heparin.

In summary, our hypothesis emphasizes electrostatic interaction between SARS-CoV-2 (especially RBD variants) and HSPGs, in the entry into host cells, but also predicts heparin's potential in the invasiveness blockage to virulent variants of SARS-CoV-2, which would be informative for drug design and vaccine development. The hypothesis provides an unproven theory for preventing and controlling the COVID-19 pandemic caused by present and new virus variants.

## Materials and methods

### Structures of S protein and mutated S protein

The structure of the SARS-CoV-2 S Protein (PDB ID:7DWZ and 7DF3) and RBD-hACE2 receptor complex (PDB ID: 6M0J) were collected from the Protein Data Bank (https://www.rcsb.org) (10, 11, 70). Based on Global Initiative on Sharing All Influenza Data (GISAID) Database (https://www.gisaid.org) available on 26 December 2021, we summarized information about the most common mutations in the RBD domain of VOCs and VOIs. Structures with the mutated residues were predicted by using the Rotamers tool of UCSF (University of California San Francisco) Chimera v1.15 based on the WT SARS-CoV-2 S Protein and hACE2-RBD complex (71). We used the Dunbrack backbone-dependent rotamer library to model rotamers of substituted residue, then chose the one with the highest probability for subsequent analysis (72).

### Electrostatic potential analysis

3-dimensional surfaces of the molecular electrostatic potential (MEP) were generated on PyMOL using the APBS (73, 74). An electrostatic potential map of the protein surface was performed with a potential range from −5.0 kT/e (red) to +5.0 kT/e (blue). All structure models' images were performed with PyMOL v2.4.2.

## Data availability statement

The original contributions presented in the study are included in the article/supplementary material, further inquiries can be directed to the corresponding author.

## Author contributions

ZZ drafted the paper and searched the literature, performed structural analyses, prepared figures, and edited and revised the manuscript. JZ did the literature search, edited and revised the manuscript, and helped prepare figures. JW put forward the hypothesis, conceptualized, supervised the execution of the work, and critically revised the manuscript. All authors contributed to the article and approved the submitted version.

## Funding

This work was supported by grants from the National Natural Science Foundation of China (91957124) and the Program of Shanghai Academic/Technology Research Leader (20XD1403200).

## Conflict of interest

The authors declare that the research was conducted in the absence of any commercial or financial relationships that could be construed as a potential conflict of interest.

## Publisher's note

All claims expressed in this article are solely those of the authors and do not necessarily represent those of their affiliated organizations, or those of the publisher, the editors and the reviewers. Any product that may be evaluated in this article, or claim that may be made by its manufacturer, is not guaranteed or endorsed by the publisher.
